# Coaching and Education for Diabetes Distress (CEDD): Protocol for a Randomized Controlled Trial

**DOI:** 10.2196/12166

**Published:** 2019-04-02

**Authors:** Charles C Chima, Jason L Salemi, Mohamad A Sidani, Roger J Zoorob

**Affiliations:** 1 Department of Population Health Science John D Bower School of Population Health University of Mississippi Medical Center Jackson, MS United States; 2 Department of Family and Community Medicine Baylor College of Medicine Houston, TX United States

**Keywords:** diabetes mellitus, type 2, diabetes distress, coaching, health coaching, counselling, self care, behavioral medicine, health psychology, health education, primary care

## Abstract

**Background:**

Diabetes distress (DD), a type of psychological distress specific to people with diabetes, is strongly associated with difficulties in performing self-care and inability to meet glycemic targets. Despite increased recognition of the need to manage DD, interventions that are both feasible and effective for reducing DD in routine care settings are not yet known. A pilot study showed that health coaching (HC) has some efficacy in addressing DD, but no adequately powered study has implemented a pragmatic research design capable of assessing the real-world effectiveness of HC in reducing DD.

**Objective:**

The aim of this study is to describe the rationale and design of an ongoing clinical trial, Coaching and Education for Diabetes Distress trial, that seeks to assess whether HC effectively reduces DD among primary care patients with diabetes and whether HC is more effective than an educational program targeting DD.

**Methods:**

The 2-arm randomized controlled trial is taking place at an academic family medicine practice in Houston, Texas. Both arms will receive usual care, which includes education about DD. In addition, the intervention arm will receive 8 HC sessions over a 5-month period. The primary outcome measure is reduction in DD over a 6-month period. Additional outcome measures include changes in hemoglobin A_1c_ and self-care practices (medication-taking, dietary, and physical activity behaviors).

**Results:**

As of March 2019, screening and recruitment are ongoing, and the results are expected by July 2020.

**Conclusions:**

HC is feasible in primary care and has been successfully applied to improving chronic disease self-management and outcomes. This study will provide evidence as to whether it has significant value in addressing important unmet psychological and behavioral needs of patients with diabetes.

**Trial Registration:**

ClinicalTrials.gov NCT03617146; https://clinicaltrials.gov/ct2/show/NCT03617146 (Archived by WebCite at http://www.webcitation.org/76Va37dbO)

**International Registered Report Identifier (IRRID):**

DERR1-10.2196/12166

## Introduction

Diabetes distress (DD) is a negative emotional reaction to diagnosis of diabetes and concerns about treatment demands, risk of complications, and inadequate support [1]. DD, along with major depressive disorder (MDD) and other depressive symptomatology, constitutes significant emotional and psychological burden to people with diabetes [1,2]. Among these distinct conditions, DD is most strongly associated with difficulties in performing self-care and inability to meet glycemic targets [2-4], and it is more common than MDD [5], leading to calls for DD management to become an essential component of diabetes care [1,6].

A review by Sturt et al [7] identified 6 approaches to address types of DD that have been studied; namely, *psychological* (eg, psychotherapy), *educational* (purely informational), *educational with some behavioral component*, *psychoeducational* (education plus a psychological intervention targeting motivation or affect), *medications and devices*, and *care management or case management* [7]. Of these, psychoeducational approaches were the only approaches found to significantly reduce DD compared with controls. Similarly, a subsequent review [8] found that DD showed improvement following interventions that target both emotion (motivational strategies) and cognition (enlightenment or education).

Psychoeducational interventions that target emotion and cognition are a broad category that includes strategies such as health coaching (HC) and psychotherapy plus education; therefore, it is necessary to identify emotion-cognition interventions that are both feasible and effective for reducing DD in routine care settings. HC is an evolving profession that helps individuals achieve sustainable behavioral change through a growth-promoting relationship that elicits autonomous motivation and improves knowledge, self-efficacy, and self-regulation [9]. HC is particularly important to diabetes care as it has the potential to address both DD and self-care.

The American Diabetes Association (ADA) recommends that providers “routinely monitor people with diabetes for DD, particularly when treatment targets are not met...” [10]. However, ADA’s recommendations for dealing with DD (ie, education, referral to a behavioral health provider, or referral to a mental health specialist) are varied and confusing [10]. On the other hand, the Canadian Diabetes Association [11] recommends an approach that is consistent with HC as defined by Olsen [12] and Wolever et al [13]. Even though some interventions have incorporated elements of coaching [7,8], only 1 study has explicitly sought to address DD as a primary outcome using HC [14]. This study found HC to be effective in addressing DD, but the finding has limited generalizability because of methodological limitations such as a small sample size and restriction to a single gender [14]. A recent trial that combined diabetes self-management education with some elements of HC and additional support was successful in addressing DD, but the intervention was too intensive to be feasible and scalable in routine primary care [15]. We proposed to conduct a randomized controlled trial (RCT) to fill the void in the available evidence on the real-world effectiveness of HC on DD. The primary aims of the study are to assess whether education alone is sufficient to address DD and whether HC has additional benefits in addressing DD beyond the effects of education. As coaching services are not typically reimbursed by insurance, cost has been a barrier to greater integration of HC in chronic illness care [16]. Therefore, one of the secondary aims of the study is to assess the willingness of patients with DD to pay for HC. The other secondary aim is to find out whether HC has additional impact on diabetes self-care and hemoglobin A_1c_ (HbA_1c_), beyond any effects observed in the group that receives only an educational program targeting DD.

## Methods

### Study Design and Setting

A 2-arm, parallel RCT is being conducted among adults with diabetes who are not meeting glycemic targets (defined as HbA_1c_ ≥8.0), at an academic family medicine clinic in Houston, Texas. The overarching goal of the study is to use a pragmatic approach to assess the effectiveness of HC in addressing DD in real-world primary care settings, in which most diabetes care is coordinated and monitored. Therefore, participants randomized to the control group will receive the current standard of care for patients not meeting glycemic targets at the study clinic as summarized in Textbox 1.

Both arms of the study (control and intervention) will receive the standard of care detailed in Textbox 1. In addition, the intervention arm will receive 8 HC sessions to address DD. The HC sessions will be held biweekly in the first 3 months (6 sessions), followed by monthly sessions in the fourth and fifth month.

**Standard of care for diabetes at the study site.**

- Quarterly follow-up visits during which patients receive ongoing, comprehensive assessment and treatment. When patients with diabetes whose HbA_1c_ is 8.0 or above exceed a 4-month period without a visit, they are contacted to schedule a follow-up appointment.
- Pharmacologic treatment of diabetes and comorbidities
- Behavioral management—diabetes self-management education and support (DSMES), that covers lifestyle modification counseling (nutrition and physical activity), medication-taking behaviors, foot care, how to monitor blood glucose (especially for those on insulin), and how to administer insulin if on insulin. This ongoing DSMES is provided through different means as follows:
  - Directly by primary care providers
  - Via health education handouts in the form of printed materials and via an electronic patient portal
  - By referral to a registered dietitian located at the clinic
- Referral to an ophthalmology clinic for annual eye check-up
- Psychological management—as part of a newly developed quality improvement project, and in line with the 2018 American Diabetes Association’s Standards of Medical Care in Diabetes [10], the clinic has implemented psychological screening (depression and diabetes distress) for patients not meeting their glycemic targets (defined as HbA_1c_ ≥8.0). The plan of action is as follows:
  - Patients who screen positive for depression (using the Patient Health Questionnaire) [17] will be referred to colocated mental health providers.
  - A diabetes distress (DD)-specific education program has been developed for patients with DD and is being piloted through this study (Multimedia Appendix 1). This education program is oriented around the 4 domains of DD—regimen distress, emotional burden, interpersonal distress, and physician distress. Patients are provided feedback on DD screening results and are advised on ways to deal with the areas of greatest distress. Screening for DD is done using the 17-item DD scale (DDS17) [18].

### Description of the Coaching Intervention for Patients With Diabetes Distress

#### Coaching Approach

Even as HC is becoming more common in primary care, there is a wide and varied understanding of what constitutes HC [12]. For the purposes of this study, the authors use the term HC to refer to an intervention with a goal of facilitating health behavior change, conducted by a health care professional (eg, registered nurse, registered dietitian, medical assistant, clinical psychologist) who has additional training and certification as a health coach. Thus, interventions carried out by peers or lay people will not meet this standard. The authors align with the conceptualization of HC as articulated in the reviews by Olsen [12] and Wolever et al [13] and reflected in the following definition:

A patient-centered approach wherein patients at least partially determine their goals, use self-discovery or active learning processes together with content education to work toward their goals, and self-monitor behaviors to increase accountability, all within the context of an interpersonal relationship with a coach. The coach is a healthcare professional trained in behavior change theory, motivational strategies, and communication techniques, which are used to assist patients to develop intrinsic motivation and obtain skills to create sustainable change for improved health and well-being.

12

Interventions for alleviating DD need to address 2 issues: the first is helping the patient express his or her feelings about diabetes, and the second is to work with the patient to figure out what can be done to meet the patient’s needs [19]. Coaching techniques suitable for achieving the first objective include building a growth-promoting relationship with the client, establishing trust and rapport through the application of 3 core coaching skills, namely mindful listening, open-ended inquiry, and perceptive reflections [20], and helping clients handle negative emotions through an empathy protocol such as nonviolent communication [21]. To address the second objective of meeting the patient’s needs, the coach will help participants elicit self-motivation, build self-efficacy, and journey through a process of change to reach their desired goals, relying on techniques such as exploring and amplifying the best in the client through appreciative inquiry [22] and building self-efficacy through motivational interviewing [23].

The abovementioned techniques for addressing the 2 issues in DD provide the overall framework that the coach will work with to help participants address DD and overcome the underlying challenges contributing to their distress. Among these techniques, motivational interviewing is probably the best described and standardized, and it has been demonstrated to be effective in many settings [24-28].

A true HC approach recognizes that each patient is unique, focuses on meeting the needs of the patient, and emphasizes relationship development between the coach and client [12,13]. As each participant’s needs and values will be unique, the coach will tailor the intervention to the needs of each participant while staying within the overall coaching framework described above. This approach of not restricting the intervention to a rigid protocol is in keeping with the understanding that flexibility within the overall coaching paradigm is key to successful coach-client collaboration [12,13].

All coaching sessions will be delivered over the phone (ie, telecoaching) to minimize inconvenience, transportation time, and cost demands to participants. Telecoaching has been previously shown to be highly acceptable, low-cost, and effective in increasing autonomy and self-efficacy among adult patients with diabetes [29].

#### Participant Assessment

Before the first coaching session, the participant will fill out the diabetes distress scale (DDS) [18] and a subset of the Summary of Diabetes Self-Care Activities questionnaire [30] focused on medication-taking, dietary, and physical activity behaviors. Only patients with significant DD (DDS score ≥2.0) will be included in this study. The health coach will review the DDS report to obtain information on the specific type of distress (eg, regimen distress) and the primary sources of distress, that is, the specific things about diabetes management that are upsetting to that particular person (eg, not feeling motivated to keep up with diabetes self-management; see Table 1).

#### First Coaching Session

At the first coaching session, building on the findings from the participant assessment, the coach will probe further to identify possible targets for the coaching intervention (Table 1). The targets define the specific goals related to DD or the underlying stressors that the participant would like to resolve or achieve at the end of the intervention. As necessary, the coach might also explore participant’s priorities, confidence, and readiness to change concerning different aspects of diabetes self-management. The coach then explores the participant’s willingness to embark on the coaching journey, discusses the findings from the assessment, and works with the participant to identify possible coaching targets (Table 1). The participant prioritizes the coaching targets and creates goals that he or she will like to achieve and a 3-month action plan.

#### Subsequent Coaching Sessions

Participants’ progress will be reviewed at subsequent coaching sessions. Most coaching sessions focus on a specific topic, helping clients navigate and overcome emerging challenges on their change journey [9]. As necessary, the coach will tap into her or his coaching toolbox to help elevate the participant’s energy, brainstorm strategies for problem solving, develop solutions, meet challenges, and set and agree on subsequent goals [9].

For purposes of quality control, the health coach will take notes on client interactions and coaching techniques used for review with the investigators. This allows ongoing feedback to ensure appropriateness of the intervention.

### Training of Intervention Staff

HC will be provided by a registered dietitian trained and certified as a Health and Wellness Coach by one of the “Transition Programs” approved by the International Consortium for Health and Wellness Coaching [31]. The International Consortium for Health and Wellness Coaching is an organization working to standardize HC practice in the United States and across the world. DD-specific education (Multimedia Appendix 1) for participants in both arms of the study will be provided by designated medical assistants in the clinic who were trained to provide this education in a consistent manner (Textbox 1).

### Study Population, Inclusion, and Exclusion Criteria

The target population is adult patients with diabetes and DD at the study clinic. Textbox 2 summarizes the inclusion and exclusion criteria.

**Table 1:** Identifying the focus of the coaching intervention from the type and sources of diabetes distress using the diabetes distress scale.

DDS^a^ subscale	Questions (from DDS)	Possible coaching targets
Regimen distress	Not feeling confident in my day-to-day ability to manage diabetes; Feeling that I am not testing my blood sugars frequently enough; Feeling that I am often failing with my diabetes routine; Feeling that I am not sticking closely enough to a good meal plan; Not feeling motivated to keep up my diabetes self-management	Perception of low self-efficacy for diabetes self-care; Perception of low motivation for diabetes self-care; Perception of lack of success in following diabetes self-care plan
Emotional burden	Feeling that diabetes is taking up too much of my mental and physical energy every day; Feeling angry, scared, depressed, or a mixture of these feelings when I think about living with diabetes; Feeling that I will end up with serious long-term complications, no matter what I do; Feeling that diabetes controls my life; Feeling overwhelmed by the demands of living with diabetes	Low level of perceived ability to influence outcomes; Low level of perceived ability to manage emotions
Interpersonal distress	Feeling that friends or family are not supportive enough of self-care efforts; Feeling that friends or family do not appreciate how difficult living with diabetes can be; Feeling that friends or family do not give me the emotional support that I would like	Perception of low levels of social support for dealing with diabetes
Physician-related distress	Feeling that my doctor does not know enough about diabetes and diabetes care; Feeling that my doctor does not give me clear enough directions on how to manage my diabetes; Feeling that my doctor does not take my concerns seriously enough; Feeling that I do not have a doctor who I can see regularly enough about my diabetes	Perception of dissatisfaction with the quality of patient-provider communication; Perception of the need to seek a better or regular provider

^a^DDS: diabetes distress scale.

**Inclusion and exclusion criteria for the Coaching and Education for Diabetes Distress trial.**

Inclusion criteria

- Has had a diagnosis of type 2 diabetes for at least 6 months
- Aged 18 to 75 years
- Most recent hemoglobin A_1c_ taken within 30 days was 8.0 or above
- At least a moderate diabetes distress, operationalized as a mean score of 2.0 or more on the 17-item diabetes distress scale [32]

Exclusion criteria

- Moderately-severe to severe depression: Patient health questionnaire-9 score 15 or above [17]
- Other severe mental health disorder (eg, Alzheimer’s or schizophrenia)
- Current pregnancy
- Severe diabetes complications or functional deficits (eg, kidney failure requiring dialysis, amputation, or blindness)

### Study Arm Assignment

**Figure 1:**
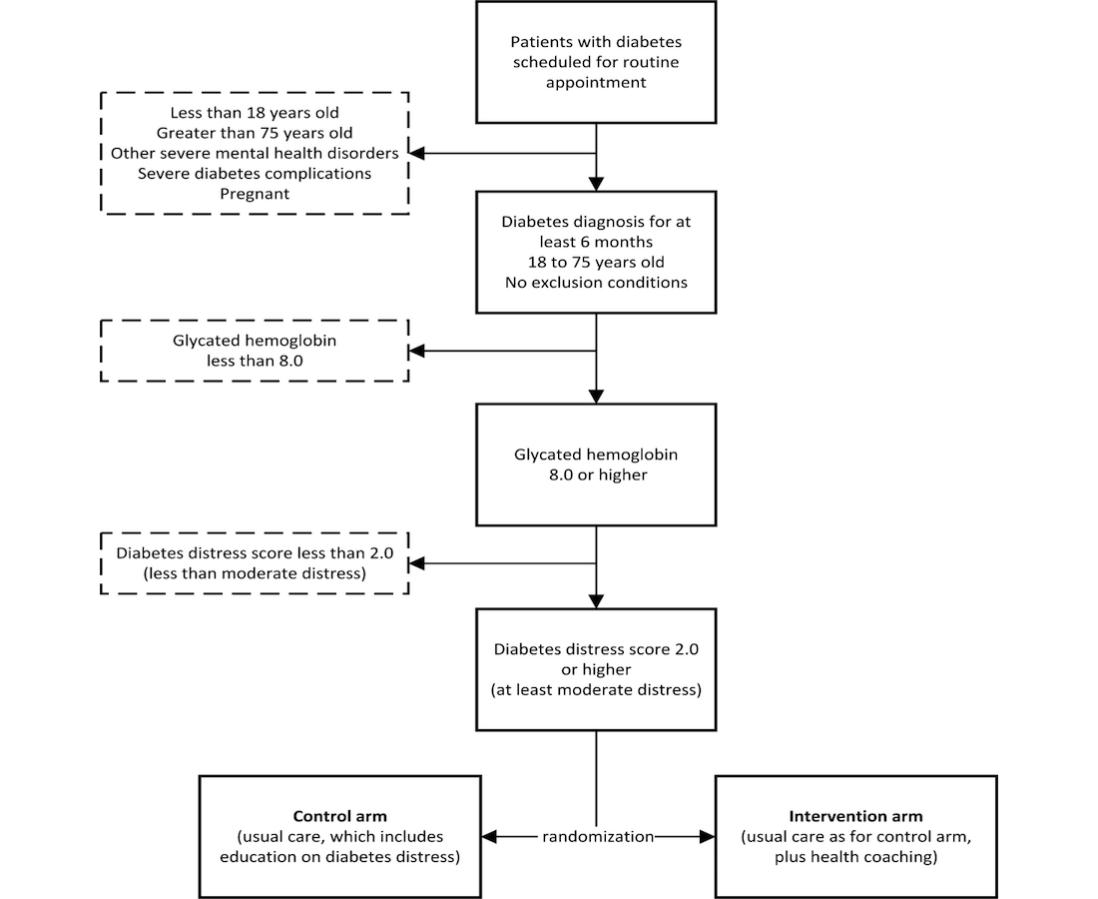
Screening, recruitment, and randomization process for Coaching and Education for Diabetes Distress trial.

Figure 1 outlines the screening, recruitment, and randomization process for the study. A random sorting randomization algorithm will be implemented using PASS software version 15.0.3 (NCSS LLC, Kaysville, UT) to generate a randomization list that will dictate assignment of patients into either the intervention or control (usual care) arm. To ensure the algorithm results in the desired group sample sizes, the program search is conducted by creating a randomization list using the user-specified randomization algorithm and then looking at the final sample sizes. If the sample sizes do not match the target sample sizes for all groups, then the list is discarded and the algorithm is restarted. This process continues until a list with the exact sample sizes is found.

### Sample Size Determination

**Figure 2:**
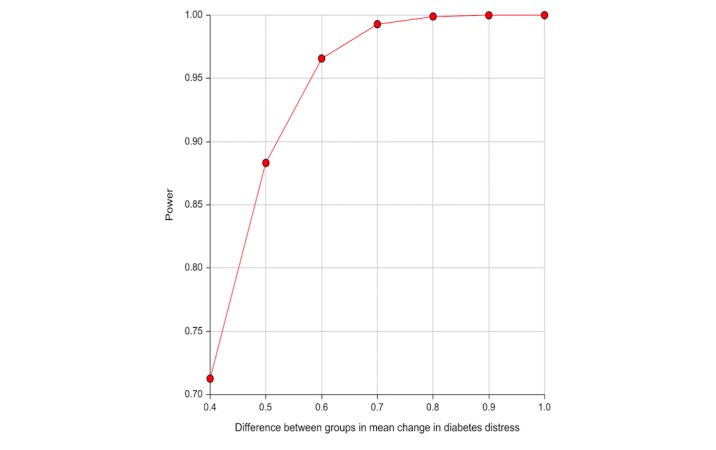
Statistical power of Coaching and Education for Diabetes Distress trial to detect effect sizes from 0.4 to 1.0.

The study requires a total sample of 156 participants, 39 in the intervention arm and 117 in the control arm. Calculations were based on the following assumptions: a power of 80%, alpha (type I error rate) of 5%, an effect size (difference in mean change in DD between the 2 groups) of 0.5, which is considered both clinically significant and at least of moderate magnitude [7], a DD SD of 0.89 and 0.81, respectively, at baseline and at follow-up [15], a correlation (ρ) of 0.5 between a pair of observations made longitudinally on the same subject, and accounting for a possible attrition rate of 20%. It has been shown that when research costs vary among treatments, it is economically efficient to randomize more participants to the cheaper arm without compromising validity [33]. Therefore, to achieve adequate statistical power within fiscal constraints, more participants will be recruited in the control arm than in the intervention arm (at a ratio of 3:1). On the basis of these assumptions and the stated sample size, this study achieves 80% statistical power to detect differences in the mean change in DD of 0.5 between the 2 groups, but it has more than 90% power to detect differences in mean changes in DD of 0.6 or higher (Figure 2).

### Measures and Data Collection

The variables that will be measured and the timeframe for data collection are summarized in Table 2. The primary outcome measure will be short-term (6 months) changes in DD score. Secondary outcome measures of interest are willingness-to-pay (WTP) for and satisfaction with HC, as well as changes in HbA_1c_ and diabetes self-care practices, specifically dietary intake, physical activity, and medication-taking behaviors.

DD, self-care behaviors, and HbA_1c_ will be assessed at 3 measurement periods, at baseline (randomization), and at the third and sixth month postrandomization. DD will be assessed using the previously validated 17-item DDS [18]. HbA_1c_ will be tested at every 3-month follow-up visit as part of usual care for patients not meeting glucose targets. Self-care practices (diet, physical activity, and medication-taking behaviors) will be assessed with the respective subscales of the Summary of Diabetes Self-Care Activities questionnaire [30]. Satisfaction with HC will be assessed for the intervention arm at the end of the study (sixth month) through self-administered surveys, including Likert scale-type questions and open-ended questions. WTP for HC will be assessed through an indirect survey approach, which has been shown to be preferable because of higher internal and external validity compared with other methods [34]. In such indirect approaches, participants are confronted with different attribute combinations of various products or services (eg, HC vs alternative interventions) with assigned prices, and they choose the most preferred scenarios. Specifically, we will conduct a discrete choice experiment to assess WTP.

**Table 2:** Measures and time frame for the Coaching and Education for Diabetes Distress trial.

Measure		Baseline	3 months from baseline	6 months from baseline
**Primary outcome measure**				
	Diabetes distress score [18]	X	X	X
**Secondary outcome measures**				
	Hemoglobin A_1c_	X	X	X
	Self-care behaviors (Medication-taking, dietary, and physical activity behaviors) using the Summary of Diabetes Self-Care Activities questionnaire [30]	X	X	X
	Willingness to pay for health coaching	X	—^a^	X
	Satisfaction with health coaching (intervention arm only)	—	—	X

^a^Not applicable.

### Data Analysis

The primary analysis will use repeated measures analysis of variance to compare mean change in DD, HbA_1c_, and self-care scores between baseline and months 3 and 6 for the 2 study arms. Satisfaction with HC will be reported as the proportion of respondents agreeing with different grades of a Likert scale. The open-ended questions on the HC satisfaction questionnaire will be analyzed qualitatively using thematic analysis. On the basis of the choices that the participants make in the discrete choice experiment, WTP for HC will be estimated by logistic regression models.

### Ethical Clearance

Patients eligible for the study will be offered the opportunity to enroll; however, the decision to enroll will be completely up to the patient. Patients who decline to enroll will continue to receive the usual standard of care; therefore, they will be informed that their decision to opt out will not affect their care in any way. An informed consent will be obtained from all patients who decide to participate. Ethical approval for the study has been obtained from the Institutional Review Board of the Baylor College of Medicine, Houston, Texas.

## Results

As of March 2019, screening and recruitment for the Coaching and Education for Diabetes Distress (CEDD) trial are ongoing and will continue through December 2019. The results will be expected by July 2020.

## Discussion

Over 30 million Americans (9.4% of the US population) were living with diabetes mellitus in 2015 [35]. With an estimated expenditure of US $101.4 billion in 2013, the United States spent more on managing diabetes than any other condition [36]. Furthermore, diabetes is the seventh leading cause of death [35] and the leading cause of adult-onset blindness, kidney failure, and nontraumatic limb amputations in adults [37]. Therefore, interventions that demonstrate real-world effectiveness in improving diabetes management have the potential to improve quality of life, reduce mortality, and decrease costs to patients, their families, and society at large.

The need to address psychological and behavioral factors as essential components of comprehensive diabetes care is increasingly being recognized. However, an effective and easily scalable intervention for DD, the most common psychological barrier to diabetes management, is not yet known. Whittemore et al [15] found that HC resulted in better self-care and reduced DD, but it had no significant effect on HbA_1c_; however, their study was limited to a small sample of women who previously voluntarily participated in diabetes education at an outpatient diabetes education center. Such women might have more intrinsic motivation than a general population of primary care patients, and they certainly might have different results from a population which includes men; therefore, the findings may not be generalizable to a general population of primary care patients. This paper reports on the design of an ongoing RCT of HC and education for DD (CEDD trial) that overcomes the methodological limitations of previous studies of coaching-related interventions for DD. The findings of this study will provide targeted evidence as to whether HC has significant value in addressing important unmet psychological and behavioral needs of patients with diabetes.

As coaching services are typically not covered by medical insurance plans at the point of care, cost has been a barrier to greater integration of HC in chronic illness care [16]. Therefore, assuming that this study finds that HC is superior to distress-specific education in addressing DD, issues of payment might constitute a barrier to scalability. By also assessing the degree to which patients with diabetes would be willing to pay for HC out of pocket, this study could pave the way for mainstreaming of HC in diabetes care. These findings would have far-reaching implications for diabetes management in routine primary care settings.

## Appendix

Multimedia Appendix 1 Diabetes Distress Education Script.

## Abbreviations

ADA: American Diabetes Association
CEDD: Coaching and Education for Diabetes Distress
DD: diabetes distress
DDS: diabetes distress scale
HbA_1c_: hemoglobin A_1c_ (glycated hemoglobin)
HC: health coaching
MDD: major depressive disorder
RCT: randomized controlled trial
WTP: willingness to pay
