# Supplementary material for: Coaching and Education for Diabetes Distress (CEDD): Protocol for a Randomized Controlled Trial
Source: JMIR Res Protoc. 2019 Apr 2;8(4):e12166. doi: 10.2196/12166 (PMC6465980; doi:10.2196/12166)
Supplement: Multimedia Appendix 1 [file resprot_v8i4e12166_app1.pdf]

APPENDIX 1: DIABETES DISTRESS EDUCATION SCRIPT

Diabetes Distress Education Script (For Medical Assistants to use at Enrollment)

Having diabetes can be overwhelming at times. When it feels like too much to deal with, you may have something called diabetes distress. This is when all the worry, frustration, anger, and burnout makes it hard for you to take care of yourself and keep up with the daily demands of diabetes.

Let us go over your current diabetes distress Score. There are two parts to your score:

Part 1 is your total score.

- A score < 2 reflects little or no diabetes distress
- A score between 2 and 3 reflects a moderate level of distress, and
- A score of 3 or higher indicates a high level of distress
- Today, your score is __(*state value*)__ which indicates that you have a __(state whether *moderate or high*)__ level of overall distress. But, the overall score only gives us part of the picture. The second part of the score helps us to determine the type of distress you appear to be having.

Part 2 helps us to determine the type of stress you appear to be having.

- Your type of diabetes distress is called _______
    - *Name type of diabetes distress based on the highest scoring dom*ain: *Emotional Burden, or Regimen Distress, or Physician Distress, or Interpersonal Distr*ess.
    - *Provide more information and advice based on the handout on the next page. Give the patient a copy of the handout*.
- People in this study are expected to visit with their primary provider every 3 months. Your distress level will be reassessed at your next visit in three months to see how things are going. Please feel free to reach out to us anytime with any question.

<table>
<tr><th>Type of Diabetes Distress</th><th>Explanation</th><th>Tips for dealing with this type of distress</th></tr>
<tr><td>Emotional Burden</td><td>Feeling that diabetes runs your life and worried about what the future holds.</td><td>
Do not hold it in: talk to family members, friends, a counselor, or other people with diabetes.
Put your feelings into words and express yourself: talking about how diabetes feels is not necessarily whining or complaining; it is sharing what is going on internally in ways that inform others and in ways that force you to articulate your concerns verbally. Talking it out can help you gain perspective, identify specific aspects of self-management that have become problematic, and make plans to address each aspect of the problem in a focused way
Seek out people who show understanding about your diabetes and are willing to listen without necessarily providing solutions. Telling the tale is often more helpful than finding the supposed solution.
</td></tr>
<tr><td>Regimen Distress</td><td>Feeling unsuccessful with your efforts to meet the demands of diabetes care recommendations.</td><td>
Consider additional diabetes self-management education with the dietitian in our clinic
Set small achievable goals and avoid trying to do too much at a time
Involve others: behavior change works best when not done alone.

Some ways to involve others in your diabetes management routine include

soliciting members of your household to help remind you to take medicines or monitor blood glucose levels (as long as these individuals are helpful and not critical);
walking with friends or family members at a set time each day; and
joining a physical activity program at a nearby neighborhood center or gym.

By engaging with others, it becomes more likely that your behavioral goals will be achieved and sustained, and distress will be reduced.

</td></tr>
<tr><td>Physician Distress</td><td>Feeling that you are getting a poor quality of care OR feeling that the communication between you and your provider is not going well.</td><td>
Plan your primary care appointments ahead of time and come to appointments with a list of questions/concerns.
Consider coming to appointments with a family member who might be able to help you communicate better with the doctor.
If you continue to feel concerned about the diabetes care you receive from your current provider, speak with the clinic's nurse to explore options, including the possibility of switching to a more suitable provider within the clinic.
</td></tr>
<tr><td>Interpersonal Distress</td><td>Feeling low levels of support from family or friends.</td><td>
Take time to think through your network of family and friends and identify close friends/family members you consider supportive towards your diabetes care or those you think you can rely on.

Have at least one designated person you can count on to be your cheerleader as you fight diabetes. Share as much information as possible with this person to enable her or him provide you the best support they can.

Identify close friends/family members who are not being supportive and have a talk with them to let them know that you will appreciate if they were more supportive.

Especially for close relatives like a spouse: if you think that he or she is major barrier to your diabetes control and you are unable to talk to them by yourself, consider bringing them along with you to your next visit and have the provider or nurse talk to them.

</td></tr>
</table>

Diabetes Distress Education Script (For Medical Assistants to use at Third Month Follow Up)

At the third month study follow up visit, participants are reassessed for diabetes distress, informed about their updated DD score, and given the following information from the Centers for Disease Control and Prevention as a handout on 10 Tips for Coping with Diabetes Distress (Source: Centers for Disease Control and Prevention https://www.cdc.gov/diabetes/ndep/people-with-diabetes/ten-tips-coping-diabetes-distress.html).

10 Tips for Coping with Diabetes Distress

Having diabetes can be overwhelming at times. The good news is that there are things you can do to cope with diabetes and manage stress. Here are 10 tips that can help.

1. **Pay attention to your feelings.** Almost everyone feels frustrated or stressed from time to time. Dealing with diabetes can add to these feelings and make you feel overwhelmed. Having these feelings for more than a week or two may signal that you need help coping with your diabetes so that you can feel better.

2. **Talk with your health care providers about your feelings.** Let your doctor, nurse, diabetes educator, psychologist, or social worker know how you've been feeling. They can help you problem-solve your concerns about diabetes. They may also suggest that you speak with other health care providers to get help.

3. **Talk to your health care providers about negative reactions other people may have about your diabetes**. Your health care providers can help you manage feelings of being judged by others because you have diabetes. It is important not to feel that you have to hide your diabetes from other people.

4. **Ask if help is available for the costs of diabetes medicines and supplies.** If you are worried about the cost of your medicines, talk with your pharmacist and other health care providers. They may know about government or other programs that can assist people with costs. You can also check with community health centers to see if they know about programs that help people get insulin, diabetes medicines, and supplies (test trips, syringes, etc.).

5. **Talk with your family and friends.** Tell those closest to you how you feel about having diabetes. Be honest about the problems you're having in dealing with diabetes. Just telling others how you feel helps to relieve some of the stress. However, sometimes the people around you may add to your stress. Let them know how and when you need them to help you.

6. **Allow loved ones to help you take care of your diabetes.** Those closest to you can help you in several ways. They can remind you to take your medicines, help monitor your blood sugar levels, join you in being physically active, and prepare healthy meals. They can also learn more about diabetes and go with you when you visit your doctor. Ask your loved ones to help with your diabetes in ways that are useful to you.

7. **Talk to other people with diabetes.** Other people with diabetes understand some of the things you are going through. Ask them how they deal with their diabetes and what works for them. They can help you feel less lonely and overwhelmed. Ask your health care providers about diabetes support groups in your community or online.

8. **Do one thing at a time.** When you think about everything you need to do to manage your diabetes, it can be overwhelming. To deal with diabetes distress, make a list of all of the tasks you have to do to take care of yourself each day. Try to work on each task separately, one at a time.

9. **Pace yourself.** As you work on your goals, like increasing physical activity, take it slowly. You don’t have to meet your goals immediately. Your goal may be to walk 10 minutes, three times a day each day of the week, but you can start by walking two times a day or every other day.

10. **Take time to do things you enjoy.** Give yourself a break! Set aside time in your day to do something you really love; it could be calling a friend, playing a game with your children or grandchildren, or working on a fun project. Find out about activities near you that you can do with a friend.

Remember that it’s important to pay attention to your feelings. If you notice that you’re feeling frustrated, tired, and unable to make decisions about your diabetes care, take action. Tell your family, friends, and health care providers. They can help you get the support you need.
